# Machine learning-based prediction of clinical outcomes in cervical cancer using routine hematological indices: development and web implementation

**DOI:** 10.3389/fonc.2025.1661153

**Published:** 2025-12-03

**Authors:** Gaigai Bai, Fanghua Chen, Junjun Qiu, Keqin Hua

**Affiliations:** Obstetrics & Gynecology Hospital of Fudan University, Shanghai Key Lab of Reproduction and Development, Shanghai Key Lab of Female Reproductive Endocrine Related Diseases, Shanghai, China

**Keywords:** cervical cancer, risk prediction, prognosis, machine learning, shiny

## Abstract

**Background:**

Cervical cancer prognosis critically depends on tumor invasiveness, yet existing predictive tools lack accessibility and generalizability. We aimed to develop predictive models using comprehensive hematological profiling of routine tests to assess invasiveness and survival, improving clinical decision-making.

**Methods:**

We conducted a retrospective analysis of 512 cervical cancer patients who underwent radical surgery. A panel of hematological indices was evaluated, including inflammatory markers, coagulation parameters, and metabolic indicators. Machine learning (ML) algorithms innovatively integrated with traditional regression were employed for feature selection and model development. Models were internally validated by bootstrap methods for discrimination (AUC/C-index) and calibration. Clinical utility was assessed by decision curve analysis (DCA). Web-based Shiny applications of these models were deployed.

**Results:**

Using routine hematological indices selected from ML-based methods, we identified the optimal variable set for each clinical outcome prediction model based on C-index comparisons. The multivariable analyses of these variables identified hematological parameters associated with cervical cancer progression and prognosis. TG, HGB, Eosinophil count, TCLR, and NAR acted as protective factors, while LDL, WBC, FAR, DDI, FLR, ENLR, SII and platelet count were risk factors linked to advanced disease features. In addition, Tbil and DDI were consistent risk factors for both recurrence-free survival (RFS) and overall survival (OS). The models assessed invasiveness risk and survival risk in two critical periods: pre-surgery and post-surgery. The AUC values for predicting locally advanced cervical cancer (LACC), uterine body invasion (UBI), lymph node positivity (LNP), adjuvant therapy (ADT), parauterine invasion (PUI), and vaginal invasion (VI) were 0.714, 0.781, 0.781, 0.719, 0.756, and 0.700, respectively. For OS, the pre-surgery and post-surgery models achieved C-index of 0.875 and 0.906, while the RFS models yielded 0.790 and 0.863, respectively. All models showed AUC ≥ 0.7, strong calibration, and positive net benefit on DCA. Interactive web tools were implemented based on these models.

**Conclusions:**

Comprehensive hematological profiling enables accurate prediction of cervical cancer invasiveness and survival during different decision-making periods. Our ML-enhanced, web-implemented models can enhance risk stratification and clinical decisions, particularly in resource-limited settings.

## 1 Introduction

Cervical cancer remains a significant global health burden, ranking as the fourth most common malignancy and cause of cancer-related mortality among women worldwide (1). Prognosis varies substantially depending on tumor stage, lymph node metastasis, and therapeutic response, with 5-year survival rates declining from 92% in localized disease to below 20% in metastatic cases (2). Despite advances in treatment, recurrence rates persist at 15-30% within 5 years post-treatment, underscoring the need for refined prognostic stratification (2).

In clinical practice, decision-making for cervical cancer management relies heavily on assessing key prognostic factors, including tumor stage, lymph node positivity (LNP), parauterine invasion (PUI), uterine body invasion (UBI), and vaginal invasion (VI). These factors not only guide therapeutic strategies but also predict surgical outcomes and survival. For instance, tumor stage determines surgical candidacy and forms the basis of initial treatment planning, while PUI and LNP are pivotal in guiding adjuvant treatment decisions, as recommended by current clinical guidelines (3). Moreover, these invasion-related parameters (PUI, LNP, UBI, and VI) also provide essential metrics for evaluating surgical complexity. Besides, the question of whether adjuvant therapy (ADT) is required adds complexity to clinical decision-making and often becomes a source of patient anxiety, making it a key concern in treatment discussions. Consequently, accurate assessment of these parameters becomes critical for both therapeutic planning and patient counseling.

However, despite the availability of numerous predictive tools for cervical cancer, critical gaps limit the clinical translation of existing predictive tools. First, many models depend on advanced imaging techniques and specialized expertise, which are often inaccessible in resource-limited settings (4, 5). Second, most tools lack patient-friendly interfaces, hindering their use in shared decision-making (6). Third, several hematological biomarkers (such as neutrophil-to-lymphocyte ratio) are clinically applicable, many molecular biomarkers, such as circulating tumor DNA or proteomic signatures, rely on specialized assays and remain less available in routine clinical settings (7). Additionally, many predictive tools for cervical cancer have been developed using datasets from limited geographic or institutional sources, which may not fully capture population diversity or clinical practice variations (8). These limitations highlight an urgent need for practical, accessible prediction tools that can predict multiple clinical outcomes in cervical cancer management.

Emerging evidence suggests that inflammation, nutrition, and metabolic status play critical roles in cancer progression, as demonstrated by extensive basic and clinical research (9–11). Routine clinical tests, such as complete blood count, liver and kidney function tests, and coagulation profiles, provide valuable insights into patients’ inflammatory, nutritional, and metabolic status. Notably, several derived indicators, including the neutrophil-to-lymphocyte ratio (NLR), mean platelet volume-to-platelet count ratio (MPV/PC), and lymphocyte-to-monocyte ratio (LMR), have shown significant prognostic relevance in cervical cancer (12–14). Additionally, the systemic immune-inflammatory (SII) indices derived from platelet count and NLR, have emerged as potential predictors of progression-free survival in patients receiving immunotherapy (15). Despite these advances, the translation of these findings into clinical practice remains challenging, primarily due to the difficulty in selecting representative and practical indicators from a wide array of available options.

To address these challenges, this study aimed to develop clinically meaningful predictive models using routinely collected clinical data, selected by machine learning combined with traditional methods. Specifically, we evaluated risks of locally advanced cervical cancer (LACC), PUI, UBI, LNP, VI, ADT, recurrence-free survival (RFS), and overall survival (OS) in two critical periods: pre-surgery and post-surgery for cervical cancer patients. Additionally, we created user-friendly, web-based tools to integrate these models into clinical practice. These tools will not only enhance healthcare providers’ ability to assess disease severity but also empower patients with personalized risk insights, facilitating informed decision-making and improving patient outcomes.

## 2 Methods

### 2.1 Data collection

**Figure 1:**
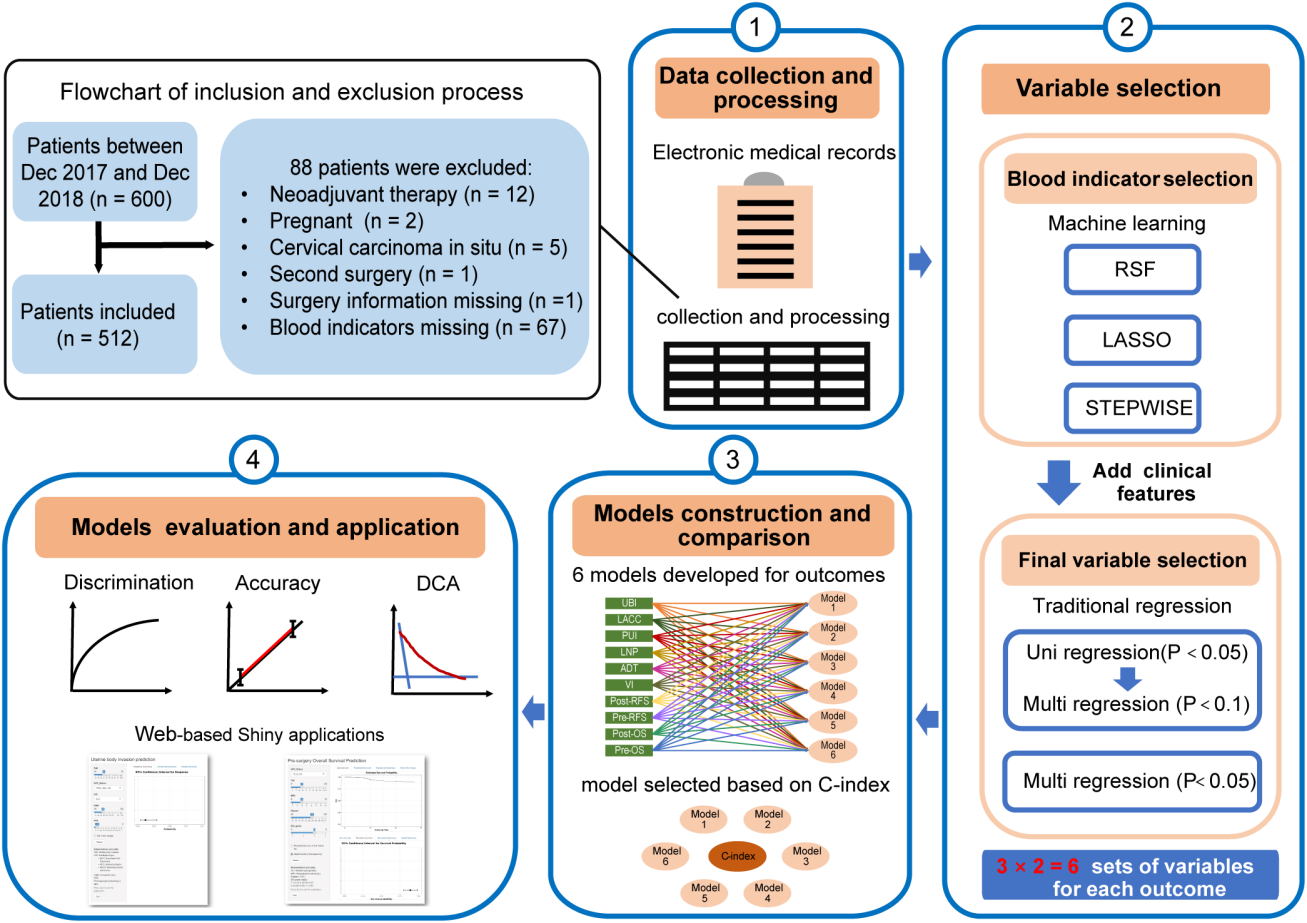
Research framework of this study.

Patient data were collected from the electronic medical records of the Obstetrics and Gynecology Hospital of Fudan University between December 2017 and December 2018. Data were extracted by two independent researchers using standardized data collection forms. The inclusion criteria were as follows: (1) diagnosis of cervical cancer confirmed by pathology; (2) patients who underwent primary radical surgery; and (3) availability of complete follow-up data in database of our center (The last follow-up time was Nov.2023). The exclusion criteria were: (1) patients who received neoadjuvant therapy; (2) patients with missing clinical information; (3) patients who had cervical carcinoma *in situ* and (4) pregnant patients or those with severe comorbidities (such as infectious diseases, liver cirrhosis, blood diseases, etc.) or combined with other cancers. A flowchart outlining the inclusion and exclusion process for patient selection is shown in **Figure 1**. This study was approved by the Ethics Committee of Obstetrics and Gynecology Hospital of Fudan University (2025-25). Informed consent was waived as the study used retrospective, anonymized data without identifiable patient information.

We collected routine blood test results, lipid profiles, liver function indices, and coagulation function indices from the hospital laboratory database. Clinicopathological factors, including age, pregnancy history, menopausal status, and family history of cancer, were obtained from inpatient medical records. The human papillomavirus (HPV) infection status of each patient was retrieved from either inpatient records or the HPV genotyping report. The American Society of Anesthesiologists Physical Status Classification System (ASA) score was extracted from anesthesia records. Histopathological results were obtained from pathology reports, and tumor staging was determined by gynecologists according to the FIGO 2018 criteria. To determine the most predictive blood biomarkers for clinical outcomes, we conducted a comprehensive literature review to identify composite indicators used in cancer prognosis prediction and subsequently calculated these indices for each individual (16–21). The calculated parameters are listed in **Supplementary Table 1**. **Figure 1** shows the study framework.

### 2.2 Variable selection for different clinical outcomes

To ensure the practicality of the predictive models, we defined the following usage scenarios: (1) predicting risk of LACC (defined as FIGO stage IB3-IVA), UBI (confirmed by pathology), LNP (confirmed by pathology), ADT (got from the medical record and followed-up data), PUI (confirmed by pathology), VI (confirmed by pathology), OS, and RFS for pre-surgery patients; and (2) OS and RFS for post-surgery patients, with known pathological results. We employed a two-step approach for variable selection, as depicted in **Figure 1**. The first step focused on selecting blood indices variables. Random Forest (RF), Least Absolute Shrinkage and Selection Operator (LASSO) regression, and Stepwise regression were used in this step to combine the strengths of machine learning and traditional statistical approaches. Random Forest identifies variables with high predictive importance in nonlinear settings, LASSO regression performs penalized selection to handle multicollinearity, and Stepwise regression selects variables based on statistical significance and information criteria (22, 23). This combined approach ensured robust and stable feature identification. The features selection process including: (1) RF, where we ranked the importance of blood indices variables using the variable importance (vimp) method and selected the more significant ones while considering collinearity determined by variance inflation factor (VIF), where VIF < 10 was considered indicative of no multicollinearity; (2) LASSO, which utilized the minimum criteria for variable selection; and (3) Stepwise Regression, which identified the optimal combination of blood indices variables for each outcome prediction according to the smallest Akaike information criterion (AIC). In the second step, we incorporated clinical pathological factors into the selected blood indices variables result from the first step. We performed both univariable + multivariable and multivariable logistic/Cox regression. For univariable + multivariable regression analyses, it is a two-step selection approach. First, candidate blood indices were screened using univariable analyses; variables with p < 0.05 were considered unlikely to be associated and were excluded. To avoid omitting variables with potential clinical relevance or borderline statistical significance, variables that met a relaxed threshold (p< 0.1) in multivariable screening were retained as candidate predictors and further entered into prediction model. In the multivariable regression, the final set of variables was selected based on a p-value threshold of < 0.05.

### 2.3 Model construction and internal validation

After the variable selection procedure, we got 6 sets of variables for each outcome (LACC, UBI, LNP, ADT, PUI, VI, pre-surgery RFS, post-surgery RFS, pre-surgery OS, post-surgery OS). Then we constructed Logistic regression models for predicting LACC, PUI, UBI, LNP, ADT and VI; Cox models for predicting survival outcomes. We selected the models based on area under the curve (AUC) of Logistic regression models and Concordance index (C-index) of Cox regression models. Models with a high AUC/C-index and relatively simpler were selected as the final models. After determining the final model of each outcome, we conducted internal validation to ensure the good performance of models. The internal validation was conducted by bootstrapping (1000 resamples) methods, composed of two parts, discrimination and accuracy, which was evaluated by Receiver Operating Characteristic (ROC) curves and calibration plots separately. An AUC ≥ 0.7 was considered acceptable discrimination. To evaluate the goodness of fit in calibration plots, the Spiegelhalter Z-test was conducted, with a p-value > 0.05 indicating no significant difference between predicted and actual probabilities. Additionally, a Brier score ranging from 0 to 0.25 was considered indicative of good model accuracy. To further assess the clinical practicality, we conducted the decision curve analysis (DCA) and plot the DCA curves. Moreover, to improve the practicality and availability of models, we deployed the models on https://www.shinyapps.io/ by using R software, so that users can input their information and get the corresponding prediction outcomes.

### 2.4 Statistical methods

All data were processed by R software (R 4.3.1). For categorical data, the numbers and frequencies were used to present it. For normal distribution continuous data, means and standard deviation (SD) were used to describe it, for Skewed distribution continuous data, median and interquartile range (IQR) were used to describe it. The survival curves were compared by Log-rank test, Benjamini-Hochberg method was used to control the false discovery rate in the group comparison. Prior to logistic and Cox regression modeling, we assessed multicollinearity using variance inflation factors (VIF < 10). In the study, statistical significance is denoted as follows: * for p < 0.05, ** for p < 0.01, *** for p < 0.001, and **** for p < 0.0001.

## 3 Results

### 3.1 Patient characteristics

A total of 600 patients were initially screened based on the inclusion and exclusion criteria. Of these, 88 were excluded, leaving 512 patients for final inclusion (**Figure 1**). The average age of the included patients was 47.61 years. Among them, 347 (66.77%) were infected with HPV types 16/18, while 32 (6.25%) were HPV negative. The majority of the patients, 452 (88.28%), reported no family history of cancer, and 161 (31.45%) were menopausal. Regarding cancer stage, 385 (75.20%) patients were classified as FIGO (2018) stage I, 55 (10.74%) as stage II, and 72 (14.06%) as stage III. Squamous cell carcinoma (SCC) was the most prevalent histological type, with 408 (79.69%) patients diagnosed with SCC. Subsequently, we analyzed the distribution of clinical outcomes of interest among the study population. Among the 512 patients, 150 (29.30%) were diagnosed with LACC. The invasion patterns were distributed as follows: UBI was observed in 51 cases (9.96%), lymph node metastasis was present in 71 patients (13.87%), and PUI was identified in 24 cases (4.69%). VI was detected in 102 patients, accounting for 19.92% of the cohort. Regarding therapeutic interventions, 194 patients (37.89%) received adjuvant treatment following primary therapy (**Supplementary Table 2**). The blood indices and composite indices of included patients were showed in **Supplementary Table 2**.

### 3.2 Survival outcomes of patients

**Figure 2:**
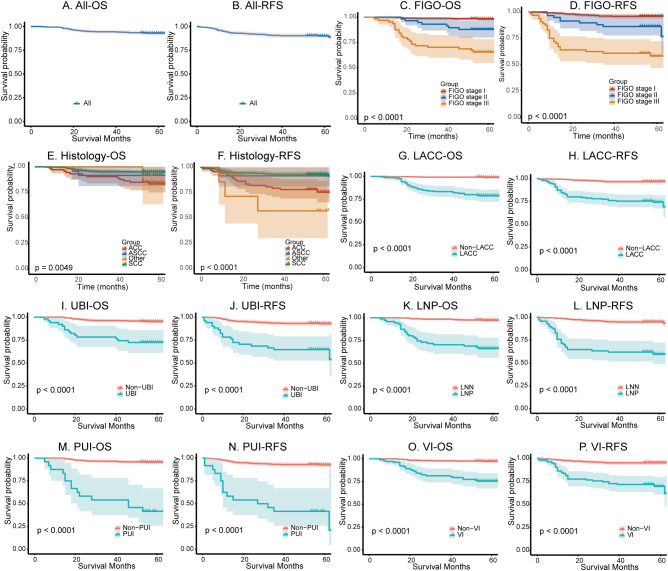
Kaplan-Meier survival analysis of cervical cancer patients. **(A, B)** Overall survival (OS) and recurrence-free survival (RFS) for the entire cohort. **(C, D)** Survival stratified by FIGO (2018) stage. **(E, F)** Survival stratified by histological type. **(G, H)** Survival in locally advanced cervical cancer (LACC) patients and Non-LACC patients. **(I, J)** Survival based on uterine body invasion (UBI). **(K, L)** Survival stratified by lymph node positivity (LNP). **(M, N)** Survival according to parauterine invasion (PUI). **(O, P)** Survival based on vaginal invasion (VI).

We subsequently evaluated the survival and recurrence outcomes of these patients. During the follow-up period, 36 patients died, and 51 experienced recurrent. The 1-, 3-, and 5-year OS rates were 99.22%, 94.53%, and 92.88%, respectively, while the corresponding RFS rates were 95.12%, 90.62%, and 90.06% (**Figures 2A, B**). Specifically, the 1, 3, 5- year OS rate and RFS rate stratified by histological type and FIGO stage were showed in **Table 1**. Prognosis varied significantly according to FIGO stage, with FIGO I patients demonstrating the most favorable outcomes, followed by FIGO II and FIGO III patients (p < 0.001) (**Figures 2C, D**). Significant differences in survival outcomes were also observed among patients with different histology types. Specifically, patients with SCC exhibited significantly better prognosis compared to those with adenocarcinoma (ACC), as evidenced by higher overall survival and recurrence-free survival rates (p = 0.0023 and p < 0.0001, respectively) (**Figures 2E, F**). Additionally, patients with LACC, UBI, LNP, PUI, or VI had significantly poorer prognosis compared to those without these characteristics (all p < 0.0001) (**Figures 2G–P**).

**Table 1:** Survival outcomes stratified by histological type and FIGO stage.

Group	OS rate			RFS rate		
	1-year OS	3-year OS	5-year OS	1-year RFS	3-year RFS	5-year RFS
ALL	99.22%	94.53%	92.88%	95.12%	90.62%	90.06%
FIGO I	100.00%	98.96%	98.34%	99.22%	96.36%	96.10%
FIGOII	100.00%	94.55%	89.09%	96.36%	87.27%	87.27%
FIGOIII	94.44%	70.83%	66.64%	72.22%	62.50%	60.00%
SCC	99.51%	95.34%	94.83%	95.83%	93.14%	93.14%
ACC	97.26%	90.41%	83.20%	94.52%	79.45%	75.39%
ASCC	100.00%	91.67%	91.67%	91.67%	91.67%	91.67%
OTHER	100.00%	100.00%	85.71%	71.43%	57.14%	57.14%

OS, Overall survival; RFS, Recurrence-free survival; SCC, Squamous cell carcinoma; ACC, Adenocarcinoma; ASCC, Adenosquamous carcinoma.

### 3.3 Variable selection and models construction

We selected the variables of each outcome according to the methods described in the second part (**Figure 1**). In first step of the blood indices variable selection, the importance ranking in the RF methods was showed in the **Supplementary Table 3**, the LASSO plots were provided in the **Supplementary Figure 1** and **Supplementary Figure 2**. Next, we proceeded to the second step, combining the clinicopathological factors with selected blood indices and obtaining the final variable set from six sets (**Supplementary Table 4**). To pick out the best variable set, we calculated the C-index for each model, the final one was the model with a higher C-index and relatively simpler variables, with their final variables was showed in **Table 2**. Additionally, our analysis revealed that lipid profiles, coagulation indices, and total bilirubin (Tbil) were prominently associated with the predictive outcomes. To further explore the prognostic relevance of hematological indices in cervical cancer, we performed uni- and multivariable regression analyses for each clinical outcome (**Supplementary Table 5**). The results revealed distinct hematological patterns associated with disease progression and prognosis. For the LACC prediction, Triglycerides (TG) and Hemoglobin (HGB) acted as protective factors, whereas Low-density lipoprotein cholesterol (LDL), White blood cell (WBC) and the ratio of Fibrinogen to Albumin (FAR) were identified as independent risk factors. In the model predicting UBI, HGB remained a protective factor, while FAR served as a risk factor. Regarding LNP prediction, D-Dimer (DDI), the ratio of Fibrinogen to Lymphocyte (FLR), the ratio of (Eosinophil × Neutrophil) to Lymphocyte (ENLR), and SII were risk factors, whereas eosinophil count, the ratio of Total cholesterol to Lymphocyte (TCLR), and the ratio of Neutrophil to Albumin (NAR) exhibited protective effects. For ADT prediction, FLR and platelet count were identified as independent risk factors. In survival analyses, both Tbil and DDI were consistent and independent risk factors for RFS and OS. Collectively, these findings indicate that a subset of routinely available hematological and biochemical parameters are significantly associated with distinct pathological characteristics and prognosis in cervical cancer.

**Table 2:** C-index of 6 variable sets for different clinical outcomes.

No.	Outcome	Model no.	Original C-index			Bootstrap C-index			Final variables
			C-index	Low 95%CI	High 95%CI	C-index	Low 95%CI	High 95%CI	
1	LACC	1	0.681	0.733	0.629	0.678	0.659	0.679	HPV infection status, TG, LDL, HGB, WBC, Lymphocyte, LDLR, FAR, ELR
		2	0.695	0.643	0.747	0.685	0.670	0.694	
		3	0.696	0.644	0.748	0.686	0.671	0.696	
		4	0.686	0.633	0.739	0.678	0.663	0.687	
		5	0.693	0.640	0.746	0.683	0.666	0.693	
		6√	0.714	0.663	0.765	0.701	0.682	0.714	
2	UBI	1√	0.781	0.716	0.846	0.765	0.726	0.784	Age, HPV infection status, Histological type, HGB, FAR
		2	0.781	0.716	0.846	0.765	0.726	0.784	
		3	0.777	0.712	0.842	0.757	0.717	0.775	
		4	0.731	0.652	0.810	0.719	0.694	0.733	
		5	0.732	0.653	0.811	0.720	0.696	0.733	
		6	0.707	0.632	0.782	0.688	0.642	0.709	
3	PUI	1	0.724	0.627	0.821	0.716	0.674	0.726	Age, Term infants, Living children, Histological type, TCLR, HDLR
		2	0.700	0.590	0.810	0.698	0.655	0.733	
		3	0.643	0.527	0.759	0.643	0.643	0.643	
		4	0.681	0.568	0.794	0.658	0.561	0.689	
		5	0.729	0.618	0.840	0.704	0.613	0.740	
		6√	0.756	0.674	0.838	0.722	0.635	0.776	
4	LNP	1	0.719	0.653	0.785	0.708	0.686	0.720	Age, TC, Eosinophil, Fibrinogen, DDI grade, FLR, TCLR, ENLR, NAR, SII
		2	0.647	0.575	0.719	0.642	0.620	0.648	
		3	0.706	0.640	0.772	0.693	0.665	0.705	
		4	0.708	0.644	0.772	0.660	0.624	0.671	
		5	0.613	0.684	0.755	0.642	0.620	0.648	
		6√	0.781	0.723	0.838	0.739	0.705	0.759	
5	ADT	1	0.703	0.657	0.749	0.694	0.678	0.704	Age, HPV infection status, Histological type, FLR, Platelet
		2	0.717	0.671	0.763	0.706	0.691	0.716	
		3√	0.719	0.673	0.765	0.707	0.690	0.718	
		4	0.678	0.629	0.727	0.669	0.654	0.679	
		5	0.709	0.662	0.756	0.698	0.682	0.708	
		6	0.719	0.673	0.765	0.702	0.684	0.715	
6	VI	1√	0.700	0.644	0.756	0.690	0.667	0.702	Menopausal status, HPV infection status, NAR, FAR
		2	0.677	0.617	0.737	0.667	0.642	0.680	
		3	0.662	0.602	0.722	0.652	0.623	0.664	
		4	0.601	0.544	0.658	0.592	0.562	0.601	
		5	0.677	0.617	0.737	0.667	0.642	0.680	
		6	0.662	0.602	0.722	0.652	0.623	0.664	
7	Post-surgery RFS	1	0.856	0.796	0.916	0.866	0.794	0.898	HPV infection status, Tbil, Lymphocyte, Platelet, DDI-grade, PLR, PUI, LNP
		2	0.856	0.796	0.916	0.866	0.794	0.898	
		3	0.856	0.796	0.916	0.866	0.794	0.898	
		4	0.839	0.775	0.903	0.857	0.768	0.875	
		5	0.791	0.784	0.798	0.803	0.716	0.839	
		6√	0.863	0.809	0.917	0.873	0.805	0.902	
8	Pre-surgery RFS	1	0.785	0.714	0.855	0.810	0.695	0.826	HPV infection status, Tbil, Lymphocyte, DDI-grade, FAR
		2	0.769	0.696	0.843	0.788	0.683	0.817	
		3	0.785	0.714	0.856	0.807	0.696	0.832	
		4	0.769	0.696	0.843	0.788	0.683	0.817	
		5	0.784	0.713	0.855	0.807	0.693	0.828	
		6√	0.790	0.722	0.858	0.808	0.707	0.837	
9	Post-surgery OS	1	0.906	0.859	0.953	0.919	0.848	0.937	HPV infection status, Tbil, DDI-grade, LNP
		2	0.906	0.859	0.953	0.919	0.848	0.937	
		3	0.863	0.796	0.930	0.874	0.790	0.914	
		4√	0.906	0.859	0.953	0.919	0.848	0.937	
		5	0.873	0.814	0.932	0.887	0.804	0.915	
		6	0.936	0.893	0.979	0.950	0.885	0.957	
10	Pre-surgery OS	1	0.836	0.757	0.915	0.846	0.752	0.901	HPV infection status, Tbil, MPV, Platelet, DDI-grade
		2	0.851	0.781	0.921	0.869	0.773	0.895	
		3	0.848	0.783	0.913	0.854	0.779	0.903	
		4√	0.875	0.824	0.926	0.887	0.814	0.911	
		5	0.848	0.783	0.913	0.854	0.779	0.903	
		6	0.899	0.847	0.951	0.915	0.839	0.929	

### 3.4 Performance of models

**Figure 3:**
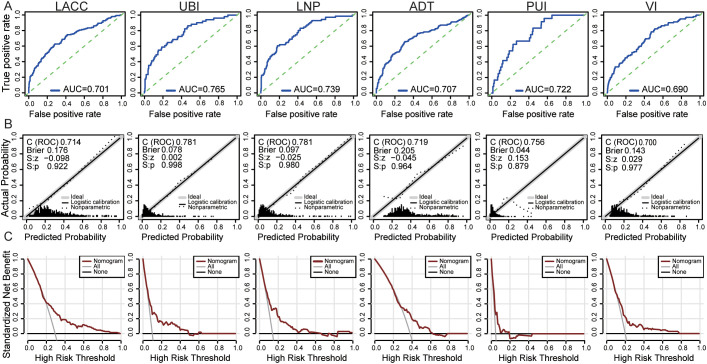
The performance of Logistic regression models. **(A)** The receiver operating characteristic (ROC) curve, **(B)** calibration curve and **(C)** decision curve analysis (DCA) plot of the models predicting locally advanced cervical cancer (LACC), uterine body invasion (UBI), lymph node positivity (LNP), adjuvant treatment (ADT), parauterine invasion (PUI) and vaginal invasion (VI).

Subsequently, we performed internal validation of models by bootstrap methods (1,000 resamples). The AUC of the model predicting LACC, UBI, LNP, ADT, PUI were 0.701, 0.765, 0.739, 0.707 and 0.722 respectively, which showed these models can discriminate the corresponding outcomes well. The model predicting VI showed moderate discrimination with the AUC = 0.690 (**Figure 3A**). To evaluate the predictive accuracy, we depicted calibration plots, which showed the high fit of the calibration curves and ideal curves (all p > 0.05). Furthermore, the Brier score of the models ranged from 0.044 - 0.205 indicating a high accuracy (**Figure 3B**). Then, the DCA was used to evaluated the clinical practicality of the models. As the DCA plots showed, these models can provide potential clinical benefits, as a significant net benefit was observed across a wide range of threshold probabilities (**Figure 3C**).

**Figure 4:**
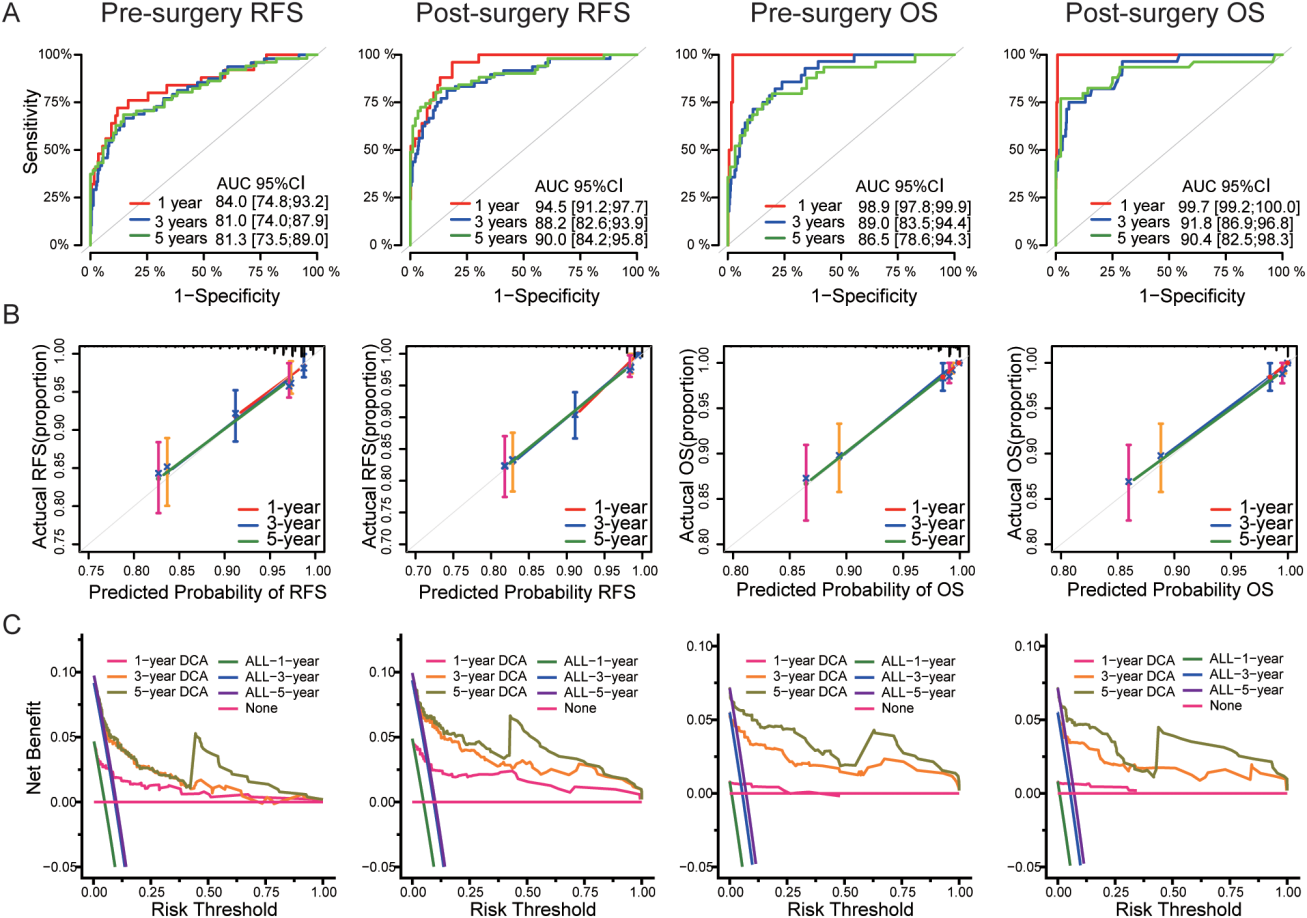
The performance of Survival predicting models. **(A)** The receiver operating characteristic (ROC) curve **(B)** calibration curve and **(C)** decision curve analysis (DCA) plot of survival predicting models.

For the survival and recurrence prediction models, the results demonstrated that four models exhibited good discrimination ability. Specifically, the 1-, 3-, and 5-year AUC values were as follows: 0.840, 0.810, and 0.813 for the model predicting RFS for pre-surgery patients; and 0.945, 0.882, and 0.900 for the model predicting RFS for post-surgery patients; 0.989, 0.890, and 0.865 for the model predicting OS for pre-surgery patients; 0.997, 0.918, and 0.904 for the model predicting OS for post-surgery patients (**Figure 4A**). The calibration curves demonstrated excellent agreement between the models’ predicted survival probabilities and the actual overall survival rates at 1, 3, and 5 years, indicating the models’ high accuracy (**Figure 4B**). DCA demonstrated that all four models provided significant net benefits across a range of threshold probabilities at 1, 3, and 5 years, indicating their potential clinical utility (**Figure 4C**).

### 3.5 The application of models

**Figure 5:**
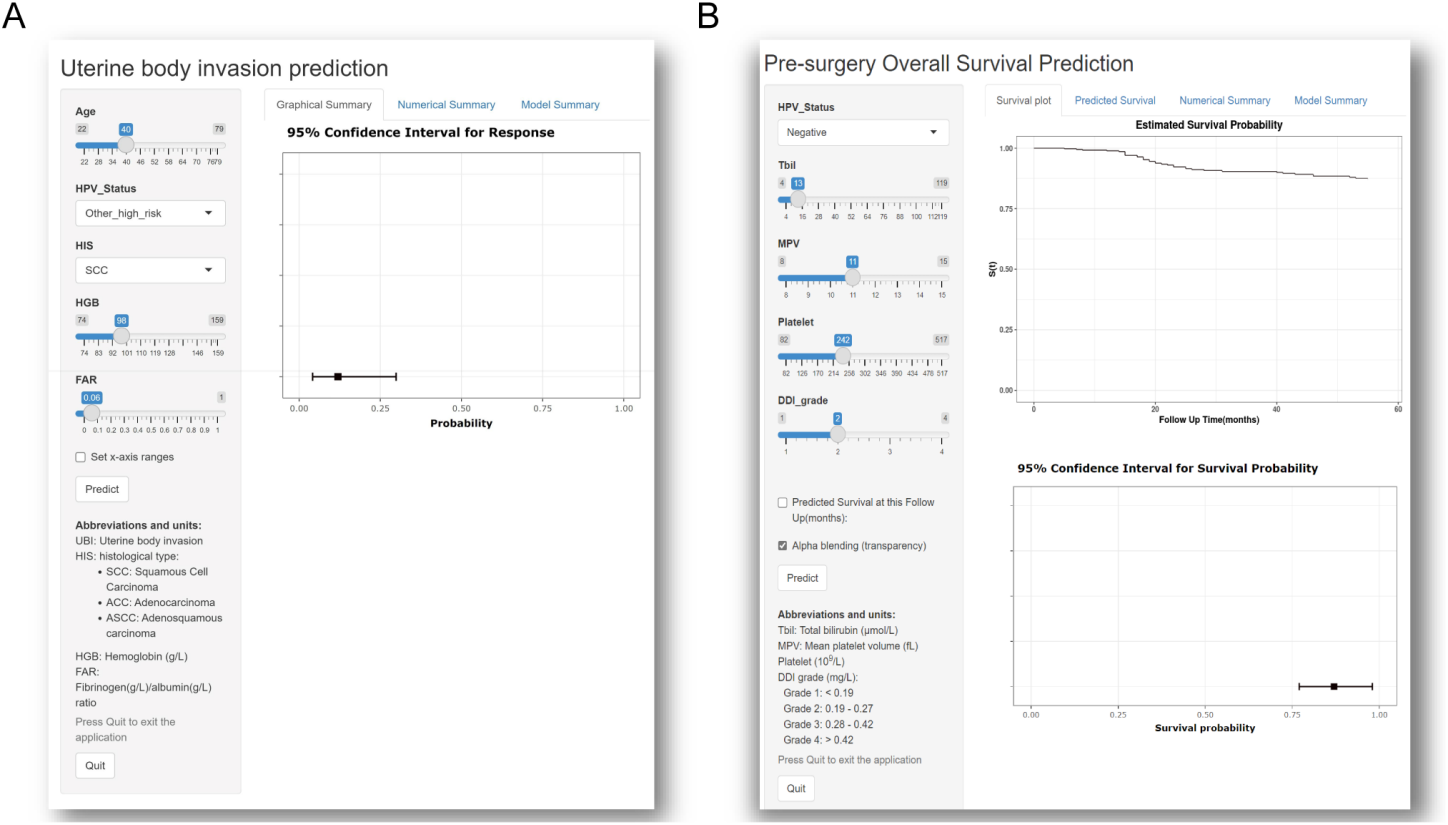
The sample interface of Logistic regression models **(A)** and survival predicting models **(B)**.

To enhance the practicality and clinical applicability of our predictive models, we deployed them as user-friendly web-based tools. These tools are accessible through the website listed in **Table 3**, where users can input their personal health data. All deployed applications are fully anonymized and do not store any patient-identifiable information, in strict accordance with institutional privacy policies and ethical standards. The web interface includes clear instructions and definitions for each input field to facilitate accurate data entry. Upon submission, users receive individualized risk estimates for the relevant clinical outcomes. For logistic regression models, predicted risks along with 95% confidence intervals (CIs) are presented both as forest plots and in numerical format. For Cox models, outputs include survival curves, forest plots at specific time points, and numerical summaries. Additionally, a summary of model performance metrics is provided on the output page to aid interpretation. Sample user interfaces are shown in **Figure 5**. These interactive tools empower patients to actively participate in their healthcare decision-making and foster more effective collaboration with their healthcare providers.

**Table 3:** Web-based Shiny applications of different outcomes prediction in cervical cancer.

Prediction outcome	Web links
LACC	https://medicalpredicitonmodel.shinyapps.io/LACC_prediction/
UBI	https://medicalpredicitonmodel.shinyapps.io/UBI_prediction/
PUI	https://medicalpredicitonmodel.shinyapps.io/PUI_prediction/
LNP	https://medicalpredicitonmodel.shinyapps.io/LNOP_prediction/
ADT	https://medicalpredicitonmodels.shinyapps.io/ADT_prediction/
VI	https://medicalpredicitonmodel.shinyapps.io/VI_prediction/
Post-surgery RFS	https://medicalpredicitonmodels.shinyapps.io/Post_surgery_RFS_predict/
Pre-surgery RFS	https://medicalpredicitonmodels.shinyapps.io/Pre_surgery_RFS_predict/
Post-surgery OS	https://medicalpredicitonmodels.shinyapps.io/Post_surgery_OS_predict/
Pre-surgery OS	https://medicalpredicitonmodels.shinyapps.io/Pre_surgery_OS_predict/

## 4 Discussion

In the context of the high incidence of cervical cancer, the complexity of its treatment, and the limited availability of applicable predictive models in clinical practice, this study developed practicable predictive models using data from routine clinical examinations. By integrating machine learning and traditional methods, we identified key blood-based variables and clinicopathological factors to construct models for predicting clinical outcomes (LACC, PUI, LNP, UBI, VI, ADT, OS, and RFS) at two critical periods: pre-surgery and post-surgery. To enhance the practical utility of these models, we created interactive Shiny apps accessible to both patients and doctors. Overall, our study not only provides valuable clinical tools but also facilitates patient understanding of their disease status, thereby improving doctor-patient communication.

With the rapid advancement of science and technology, an increasing number of predictive models have been developed to forecast critical outcomes and prognoses in cervical cancer. Traditionally, these models have primarily relied on clinical factors. For example, Guo et al. constructed a predictive model for para-aortic lymph node metastasis based on pathological features of lymph nodes, tumor size, and histological type (24). In recent years, the application of machine learning and deep learning techniques has gained traction in cervical cancer prediction. An example is the deep learning model developed by Wu et al., which utilizes MRI images to predict the risk of cervical cancer recurrence (25). Meanwhile, progress in molecular oncology has enabled the development of molecular-based predictive models. These models have been employed to forecast responses to chemotherapy and immunotherapy, as well as the likelihood of distant metastases in cervical cancer patients (26–29). Despite these advancements, only a few predictive models have been successfully integrated into clinical practice. This gap is primarily attributed to the poor accessibility and limited clinical utility of current predictive models.

Our study builds upon and extends previous research that has applied machine learning approaches to routine blood analyses for cervical cancer (30, 31). Compared with these studies, our work integrates both classical machine learning algorithms and traditional statistical methods for variable selection, providing a balanced framework that enables comprehensive screening and optimal variable combinations for model development. In addition, we incorporated clinical treatment timelines by constructing predictive models for key clinical outcomes at different stages (preoperative and postoperative), thereby enhancing clinical relevance and applicability. Moreover, the models rely on easily obtainable clinical and laboratory parameters, and we further developed an interactive web-based interface designed for both healthcare providers and patients. This tool facilitates disease severity assessment, follow-up planning, and shared decision-making, supporting more individualized patient management.

Furthermore, our study delineated several pivotal hematological markers implicated in the progression of cervical cancer. Hematological parameters such as lymphocyte, monocyte, neutrophil, and eosinophil counts, which have been previously correlated with the prognosis of colorectal, breast, and other malignancies, were reaffirmed in our research to hold prognostic significance in cervical cancer as well (19, 32, 33). Particularly, we revealed that coagulation markers (platelet count, DDI, and fibrinogen) play a substantial role in forecasting clinical outcomes in cervical cancer, including LACC, UBI, LNP, ADT, VI, RFS and OS. This association, scarcely documented in prior research, underscores the prognostic weight of a hypercoagulable state, thereby underpinning the rationale for anti-coagulation therapy in cervical cancer management. Furthermore, our investigation illuminated the cancer severity relevance of lipid metabolism indicators (TC, TG, HDL, LDL) in the trajectory of cervical cancer. Intriguingly, we identified Tbil as a risk factor for prognosis. While the prognostic utility of Tbil has been established in hepatocellular carcinoma, cholangiocarcinoma, and non-small cell lung cancer, leading to the development of associated prognostic scores (34–36), its prognostic relevance in cervical cancer remains underexplored. This gap underscores the imperative for further mechanistic and clinical inquiries into the role of Tbil in cervical cancer, to elucidate its potential as a biomarker and therapeutic target. By leveraging comprehensive clinical data, our predictive models not only identified novel blood indices, such as coagulation markers and lipid metabolism indicators, but also demonstrated their significant value in cervical cancer. These findings enhance the clinical applicability of our study, providing a robust foundation for risk stratification and personalized treatment strategies.

The limitations of this study include the following: Firstly, the data may be subject to bias due to the single-center, retrospective nature of the research. Secondly, due to limited data availability, we were unable to perform external validation. Thirdly, for the models of pre-surgery time point, the models were developed using data from patients who ultimately underwent surgery at our institution, which may introduce bias in predicting outcomes for patients who did not receive radical surgery. Future research should incorporate more comprehensive datasets to enhance the generalizability of our findings and support the development of more intelligent, user-friendly, and clinically applicable predictive models.

In summary, we developed and validated clinically applicable prediction models for cervical cancer patients across different therapeutic stages by integrating multidimensional blood indices with clinicopathological features. These models exhibited strong performance, including high AUC values, excellent calibration, and significant clinical utility as demonstrated by DCA, supporting their potential for personalized clinical decision-making. To facilitate real-world application, we deployed the models as interactive Shiny applications, enhancing accessibility and usability in clinical settings. This study not only provides a scalable predictive framework but also underscores the translational potential of routinely collected clinical data in advancing cervical cancer management.

## Data Availability

The raw data supporting the conclusions of this article will be made available by the authors, without undue reservation.
