# Supplementary material for: Machine learning-based prediction of clinical outcomes in cervical cancer using routine hematological indices: development and web implementation
Source: Front Oncol. 2025 Dec 3;15:1661153. doi: 10.3389/fonc.2025.1661153 (PMC12708249; doi:10.3389/fonc.2025.1661153)
Supplement: Supplementary file 1 [file DataSheet1.docx]

Supplementary Material

# Supplementary table

| Composite Index Calculated in this study | | | | | | | |
| --- | --- | --- | --- | --- | --- | --- | --- |
| Variables | | Calculation method | | | | | |
| NAR (10^9/g) | | | Neutrophil / Albumin | | | | |
| LMR | | | Lymphocyte / Monocyte | | | | |
| ELR | | | Eosinophil / Lymphocyte | | | | |
| PLR | | | Platelet / Lymphocyte | | | | |
| NLR | | | Neutrophil / Lymphocyte | | | | |
| FLR (g/10^9) | | | Fibrinogen / Lymphocyte | | | | |
| HDLR (mmol/10^9) | | | HDL / Lymphocyte | | | | |
| LDLR (mmol/10^9) | | | LDL / Lymphocyte | | | | |
| TCLR (mmol/10^9) | | | TC / Lymphocyte | | | | |
| TGLR (mmol/10^9) | | | TG / Lymphocyte | | | | |
| FAR | | | Fibrinogen / Albumin | | | | |
| ENLR (10^9/L) | | | (Eosinophil × Neutrophil) / Lymphocyte | | | | |
| PVPR ((fL×L)/10^9) | | | MPV / Platelet | | | | |
| SII | | | Platelet × NLR | | | | |
| SIS | | | LMR > 4.44, ALB > 40 | | | | 0 |
|  |  |  | LMR ≤ 4.44 or ALB ≤ 40 | | | | 1 |
|  |  |  | LMR < 4.44, ALB < 40 | | | | 2 |
| CONUT | Albumin (g/dL) | | | ≥3.50 | 3.00–3.49 | 2.50–2.99 | <2.50 |
|  | Score | | | 0 | 2 | 4 | 6 |
|  | Lymphocyte (/mm3) | | | ≥1600 | 1200–1599 | 800–1199 | <800 |
|  | Score | | | 0 | 1 | 2 | 3 |
|  | TC (mg/dL) | | | ≥180 | 140–179 | 100–139 | <100 |
|  | Score | | | 0 | 1 | 2 | 3 |
|  | CONUT = Add scores above | | | | | | |

**Supplementary Table 1** Composite indicators in this study.

Abbreviations: TC = Total cholesterol, LDL = Low-density lipoprotein cholesterol, HDL = High-density lipoprotein cholesterol, TG = Triglycerides, MPV = Mean platelet volume, ALB = Albumin, SII = Systemic immune inflammation index, SIS = Systemic inflammation score, CONUT = Controlling nutritional status score

**Supplementary Table 2** Baseline characteristics of included patients.

| Included patients (n = 512) | | |
| --- | --- | --- |
| Clinicopathological factors | | |
| Age | | 47.61±9.75 |
| HPV status | HPV 16/18 + | 347 (67.77%) |
|  | Other high risk + | 57 (11.13%) |
|  | Unknown type + | 53 (10.35%) |
|  | Negative | 32 (6.25%) |
|  | Unknown status | 23 (4.49%) |
| Family History | Yes | 60 (11.72%) |
|  | No | 452 (88.28%) |
| Menopause | Yes | 161 (31.45%) |
|  | No | 351 (68.55%) |
| FIGO (2018) stage | I | 385 (75.20%) |
|  | II | 55 (10.74%) |
|  | III | 72 (14.06%) |
| Histology | SCC | 408 (79.69%) |
|  | ACC | 73 (14.26%) |
|  | ASCC | 24 (4.69%) |
|  | Other | 7 (1.37%) |
| ASA Physical status | 1 | 290 (56.64%) |
|  | 2 | 219 (42.77%) |
|  | 3 | 3 (0.58%) |
| LACC  (IB3-IVA) | Yes | 150 (29.30%) |
|  | No | 362 (70.70%) |
| UBI | Yes | 51 (9.96%) |
|  | No | 461 (90.04%) |
| Lymph node | Positive | 71 (13.87%) |
|  | Negative | 411 (80.23%) |
| ADT | Yes | 194 (37.89%) |
|  | No | 318 (62.11%) |
| PUI | Yes | 24 (4.69%) |
|  | No | 488 (95.31%) |
| VI | Yes | 102 (19.92%) |
|  | No | 410 (80.08%) |
| Liver function indices | | |
| AKP (U/L) | | 75 (62-93) |
| Tbil (umol/L) | | 11 (9-14) |
| ALB (g/L) | | 45 (43-47) |
| Lipid profiles | | |
| TC (mmol/L) | | 4.68 (4.07-5.31) |
| LDL (mmol/L) | | 2.99 (2.61-3.39) |
| HDL (mmol/L) | | 1.13 (0.97-1.31) |
| TG (mmol/L) | | 1.21 (0.89-1.72) |
| Routine blood test | | |
| HGB (g/L) | | 129 (122-135) |
| Basophil (10^9/L) | | 0.024 (0.01-0.03) |
| MPV (fL) | | 10.80 (10.10-11.50) |
| PDW (fL) | | 12.8 (11.5-14.50) |
| Platelet (10^9/L) | | 237.5 (203.0-281.2) |
| WBC (10^9/L) | | 6.04 (5.05-7.23) |
| Eosinophil (10^9/L) | | 0.07 (0.04-0.12) |
| Lymphocyte (10^9/L) | | 1.67 (1.38-2.00) |
| Monocyte (10^9/L) | | 0.37 (0.30-0.46) |
| Neutrophil (10^9/L) | | 3.81 (2.85-4.86) |
| Coagulation function indices | | |
| DDI grade* (mg/L) | 1 | 218 (42.58%) |
|  | 2 | 101 (19.73%) |
|  | 3 | 91 (17.78%) |
|  | 4 | 102 (19.92%) |
| Fibrinogen (g/L) | | 2.50 (2.20-2.80) |
| Composite index | | |
| NAR (10^9/g) | | 0.085 (0.065-0.111) |
| LMR | | 4.54 (3.60-5.58) |
| ELR | | 0.039 (0.022-0.069) |
| PLR | | 143.64 (112.35-179.92) |
| NLR | | 2.29 (1.68-3.09) |
| FLR (g/10^9) | | 1.53 (1.23-1.89) |
| HDLR (mmol/10^9) | | 0.68 (0.56-0.85) |
| LDLR (mmol/10^9) | | 1.77 (1.49-2.19) |
| TCLR (mmol/10^9) | | 2.80 (2.35-3.41) |
| TGLR (mmol/10^9) | | 0.75 (0.54-1.05) |
| FAR | | 0.056 (0.049-0.064) |
| ENLR (10^9/L) | | 0.15 (0.08-0.27) |
| PVPR ((fL×L)/10^9) | | 0.045 (0.037-0.055) |
| SII | | 547.7 (369.0-777.1) |
| SIS | 0 | 253 (49.41%) |
|  | 1 | 240 (46.88%) |
|  | 2 | 19 (3.71%) |
| CONUT | 0 | 164 (32.03%) |
|  | 1 | 182 (35.55%) |
|  | 2 | 111 (21.68%) |
|  | 3 | 42 (8.20%) |
|  | 4 | 11 (2.15%) |
|  | 5 | 2 (0.39%) |

Abbreviations: HPV = Human papillomavirus, ASA = American Society of Anesthesiologists Physical Status Classification System,‌ LACC = Locally advanced cervical cancer, UBI = Uterine body invasion, ADT = Adjuvant therapy, PUI = Parauterine invasion, VI = Vaginal invasion, AKP = Alkaline phosphatase, Tbil = Total bilirubin, ALB = Albumin, TC = Total cholesterol, LDL = Low-density lipoprotein cholesterol, HDL = High-density lipoprotein cholesterol, TG = Triglycerides, HGB = Hemoglobin, MPV = Mean platelet volume, PDW = Platelet distribution width, WBC = White blood cell, DDI = D-Dimer *The DDI grade was classified based on the lower detection threshold (0.19) and the 33rd and 66th percentile values of the cohort as follows: Grade 1: < 0.19, Grade 2: 0.19 - 0.27, Grade 3: 0.28 - 0.42, Grade 4: > 0.42.

# Supplementary Figures


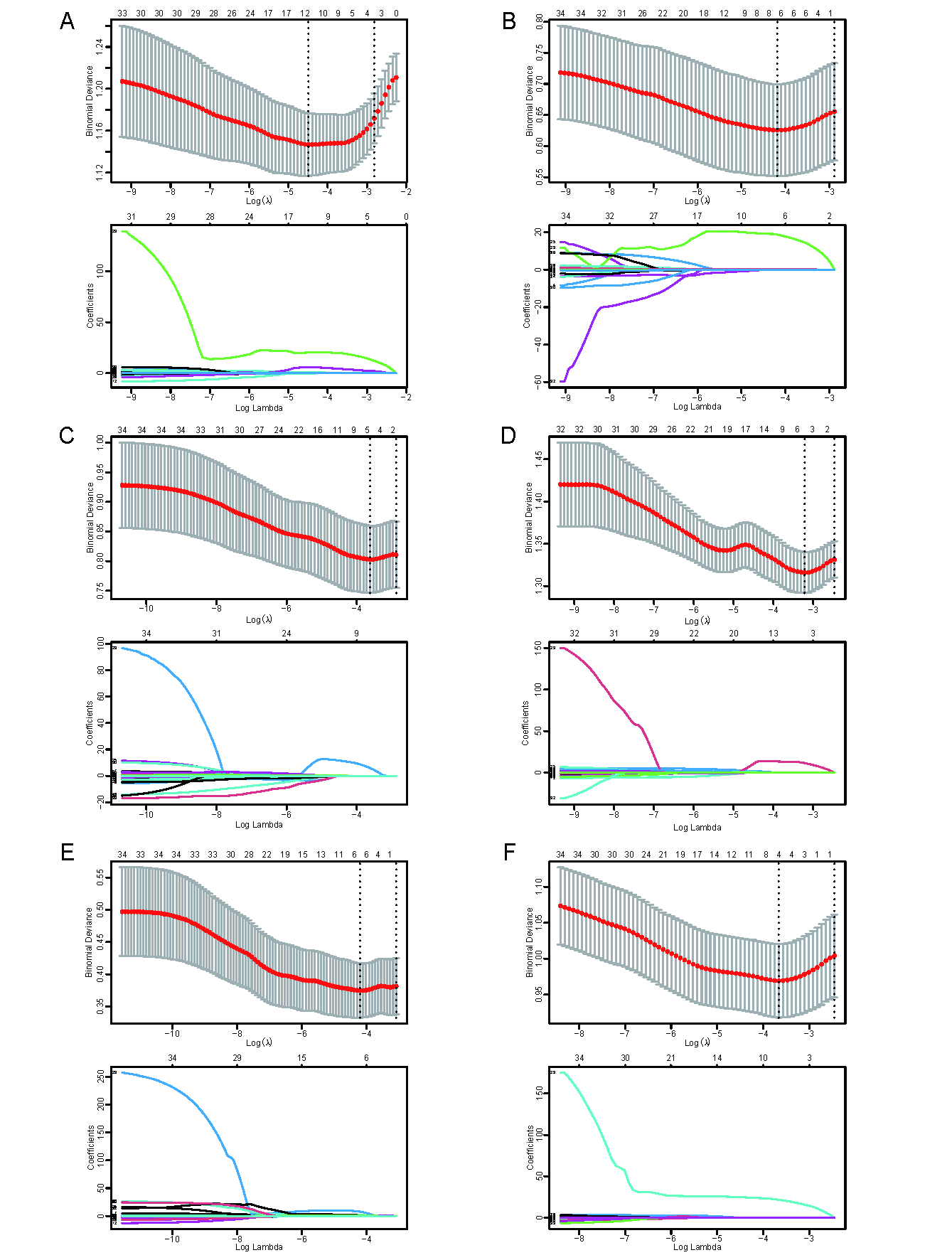


**Supplementary Figure 1.** LASSO feature selection of blood indices for Logistic model construction to predict (A) LACC, (B) UBI, (C) LNP, (D) ADT, (E) PUI and (F)VI.


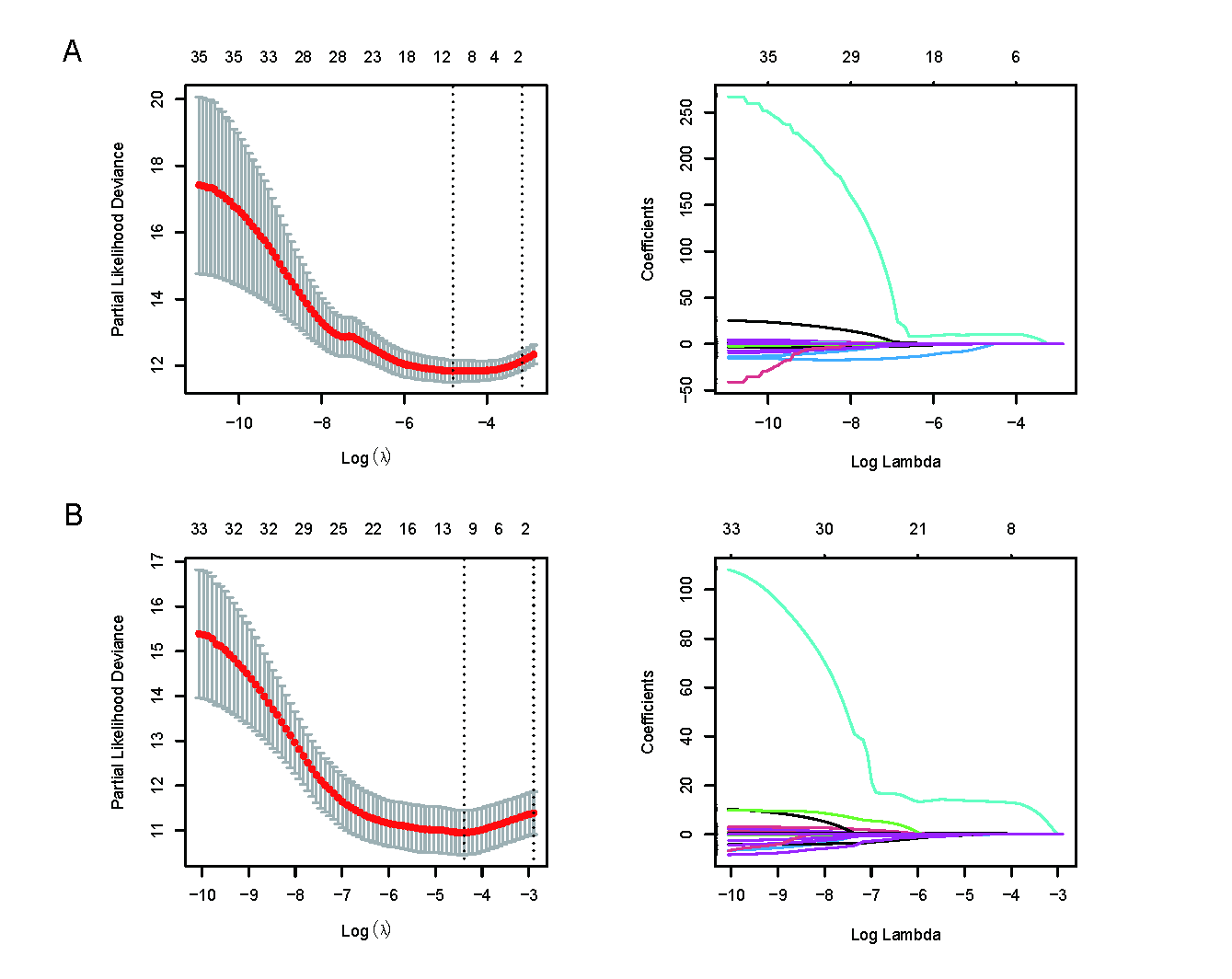


**Supplementary Figure 2.** LASSO feature selection of blood indices for survival model construction to predict (A) OS and (B) RFS.
